# Assessing the Perception of Trunk Movements in Military Personnel with Chronic Non-Specific Low Back Pain Using a Virtual Mirror

**DOI:** 10.1371/journal.pone.0120251

**Published:** 2015-03-23

**Authors:** Meyke Roosink, Bradford J. McFadyen, Luc J. Hébert, Philip L. Jackson, Laurent J. Bouyer, Catherine Mercier

**Affiliations:** 1 Centre Interdisciplinaire de Recherche en Réadaptation et Intégration Sociale (CIRRIS), Québec, Québec, Canada; 2 Department of Rehabilitation, Faculty of Medicine, Laval University, Québec, Québec, Canada; 3 Canadian Forces Health Services Group Headquarters, Directorate of Medical Policy (Physiotherapy), Valcartier Garrison, Québec, Québec, Canada; 4 Department of Radiology, Faculty of Medicine, Laval University, Québec, Québec, Canada; 5 School of Psychology, Laval University, Québec, Québec, Canada; The Hong Kong Institue of Education, HONG KONG

## Abstract

Chronic pain, including chronic non-specific low back pain (CNSLBP), is often associated with body perception disturbances, but these have generally been assessed under static conditions. The objective of this study was to use a “virtual mirror” that scaled visual movement feedback to assess body perception during active movement in military personnel with CNSLBP (n = 15) as compared to military healthy control subjects (n = 15). Subjects performed a trunk flexion task while sitting and standing in front of a large screen displaying a full-body virtual mirror-image (avatar) in real-time. Avatar movements were scaled to appear greater, identical, or smaller than the subjects’ actual movements. A total of 126 trials with 11 different scaling factors were pseudo-randomized across 6 blocks. After each trial, subjects had to decide whether the avatar’s movements were “greater” or “smaller” than their own movements. Based on this two-alternative forced choice paradigm, a psychophysical curve was fitted to the data for each subject, and several metrics were derived from this curve. In addition, task adherence (kinematics) and virtual reality immersion were assessed. Groups displayed a similar ability to discriminate between different levels of movement scaling. Still, subjects with CNSLBP showed an abnormal performance and tended to overestimate their own movements (a right-shifted psychophysical curve). Subjects showed adequate task adherence, and on average virtual reality immersion was reported to be very good. In conclusion, these results extend previous work in patients with CNSLBP, and denote an important relationship between body perception, movement and pain. As such, the assessment of body perception during active movement can offer new avenues for understanding and managing body perception disturbances and abnormal movement patterns in patients with pain.

## Introduction

Chronic pain is often associated with reports of body perception disturbances such as subjective swelling or numbness [1] and impaired position sense [2,3]. Still, the mechanisms underlying these phenomena are not well understood. Recent neurophysiological evidence points to a potential role for brain reorganization by demonstrating a relationship between clinical pain characteristics (e.g., pain intensity/duration) and brain activation patterns in the sensorimotor cortex in various pain populations (for a review see [1]). However, given the dependency of body perception disturbances on the position of the body in space [4], body perception disturbances could well be linked to abnormal integration of multimodal sensory information, rather than to the somatotopic organization of the body per se [5,6]. Moreover, it is likely that pain interacts with sensorimotor control at multiple levels in the nervous system, additionally involving peripheral, spinal and cognitive-emotional functions [7].

The assessment of body perception disturbances (or abnormal body representations [8]) can be done in various ways, but so far no gold standard has been identified. Questionnaires are available to assess perceived somatosensory abnormalities (e.g., temperature, size, pressure, and posture) [9], or body awareness [10], and can be complemented by drawings or animations of perceived disturbances [9,11,12,13]. Experimental tools usually require the estimation or judgment of certain properties of the body (i.e., relying on interoception) under various environmental circumstances (i.e., relying on exteroception). For example, the integrity of body or movement representations has been assessed by having subjects estimate the size of their body parts [14], perform motor imagery [15,16], visually recognize body-object interactions [17], or by determining the order of segments that make up a particular movement [18]. A particular aspect of body perception deals with the perceived position of one’s own body in space, which can be estimated by having subjects indicate their subjective body midline or the location of body parts in front of them [19,20,21].

In most studies, body perception and multisensory integration have been assessed under static conditions or during passive movement rather than during active movement (i.e., relying on continuous interoception). This is surprising as clinical observations indicate that body perception disturbances might also, or perhaps even solely, be observed while performing movements [7]. Therefore, movement-dependent assessments would correspond better with real-world body-environment interactions, and could offer new avenues for studying, understanding, and managing the interaction between pain, body perception disturbances and abnormal movement patterns.

One of the clinical populations in which body perception assessments during active movement could be particularly interesting is the population of patients with chronic non-specific low back pain (CNSLBP). Low back pain is the primary chronic complaint of approximately 20% of regular and 8% of reserve Canadian Armed Forces (CAF) members (CFHLI Survey 2004 Regular and Reserve Force reports), and is of increasing concern to the CAF health services, as it is affecting operational readiness. In the general population, previous work has already identified various types of static body perception disturbances in patients with CNSLBP [12,15,22,23]. Moreover, CNSLBP has been associated with reduced physical activity [24], abnormal movement patterns (for a review see [7]), impaired proprioception [3,25,26] and movement-related psychological factors such as fear-of-movement and pain-catastrophizing [27,28].

Recently, we developed a “virtual mirror” displaying a realistic full-body avatar that responds to full-body movements in all movement planes in real-time [29]. This “virtual mirror” can be used to artificially exaggerate (i.e., augment) or reduce the visual feedback on actual movements at a specific joint, while providing normal feedback at other joints. As such, this set-up offers the possibility to create a match or mismatch of visual (i.e., exteroceptive) and proprioceptive (i.e., interoceptive) information during active movement. In order to study CNSLBP, a trunk flexion task was developed. This task consists of a series of trunk flexion movements during which different levels of scaled (augmented/reduced) visual movement feedback are applied. Using a two-alternative forced choice paradigm, body perception can then be assessed during active movement by modeling a psychophysical curve. From this curve, the ability to discriminate between different levels of scaled feedback (just noticeable difference, JND) as well as the presence of perceptual biases (point of subjective equivalence, PSE) can be derived.

The objective of this exploratory study was to use this “virtual mirror” in combination with the trunk flexion task to assess body perception during active movement in military personnel with CNSLBP as compared to military healthy control (HC) subjects. We hypothesized that the ability to detect scaled visual movement feedback would be reduced in subjects with CNSLBP as compared to HC subjects, as reflected in higher JNDs and a shifted PSE.

## Materials and Methods

### Subjects

The project was performed in collaboration with the Canadian Armed Forces (CAF). A convenience sample of 16 military personnel with CNSLBP and 16 age-matched military HC subjects (aged between 18–55 years, men only to comply with the avatar’s gender) were recruited at a regional military base (sample size based on a proof-of-principle study including 10 healthy military subjects [29]). Subjects with CNSLBP were eligible when reporting persistent low back pain for at least 3 months, and when physically capable of performing repetitive trunk exercises. General exclusion criteria included non-corrected visual impairments and repeated fractures. CNSLBP-specific exclusion criteria included bilaterally radiating pain, a positive static leg raise test during the 12 weeks prior to participation, and surgery or invasive treatment of the back or spine during the past year. HC-specific exclusion criteria included recurrent low back pain, low back pain that required medical care or that restricted work or recreation during the past 2 years, chronic pain (duration ≥ 3 months) during the last 6 months prior to participation, or other medical conditions (inflammatory, neurologic, degenerative, auto-immune, psychiatric) that could interfere with performance during testing. All assessments took place at the Centre interdisciplinaire de recherche en réadaptation et intégration sociale of the Institut de réadaptation en déficience physique de Québec (IRDPQ). The project and consent procedure were approved by the institutional review board of the IRDPQ (#2013–323). All subjects received written and oral information, and provided written informed consent prior to participation. Transportation costs associated with participation were reimbursed.

### Demographic and anthropomorphic data

Age and anthropomorphic data (weight, height, and body mass index) were recorded for each subject.

### CNSLBP characteristics

For subjects with CNSLBP, a standardized form was used to collect information on the intensity (maximum and average pain intensity over the past 48 hours; numeric rating scale, NRS: 0 = no pain, 100 = worst pain imaginable), duration (time since onset of current episode of low back pain, total number of episodes), frequency (number of days per week), and location (left, right, center, alone or in combination) of pain. Additionally, information was gathered on the types of activity that increased CNSLBP, on current treatment (physiotherapy, medication), on relevant co-morbidities, and on work restrictions. Low back pain associated disability was assessed using a French version of the Oswestry Disability Index (ODI) [30,31]. The ODI’s total score ranges from 0–100%, with a score of 0–20% indicating minimal disability, and a score of 21–40% indicating moderate disability. Kinesiophobia (fear of movement) was assessed using French-Canadian versions of the 17-item Tampa Kinesiophobia Scale (TSK) [32](Université de Montréal, 2005) and the Fear of Daily Activities Questionnaire (FDAQ) [33](CIRRIS, 2014). For the TSK the total score ranges between 17–68 and a score equal to or higher than 37 is indicative of significant kinesiophobia [28]. The FDAQ assesses the average fear rating related to low back pain on 10 predefined activities based on an NRS (0 = no fear, 100 = maximal fear).

### Experimental procedure

The entire experiment was performed during a single experimental session of 2.5h. A detailed description and visual presentation of the virtual mirror system, as well as of associated experimental procedures and methodological considerations, has been published elsewhere [29].

#### Preparations and familiarization

Subjects put on a motion capture suit (OptiTrack, NaturalPoint, Corvallis, Oregon, USA) and a set of 41 reflective markers (14 mm diameter spheres) were placed over the entire body according to a standardized protocol (“HumanRTkm”, Vicon Motion Systems Ltd., Oxford, UK). After calibrating the motion capture system (12 Bonita10 camera’s, 100 Hz sampling frequency, Vicon Motion Systems Ltd., Oxford, UK), subjects were placed in the dark at a distance of 2 meters in front of a silver-coated projection screen (projection surface 3.05 m x 2.06 m), with their avatar being projected in front of them (D-flow software, Motek Medical, Amsterdam, The Netherlands). A single projector was used (Hitachi, Tokyo, Japan; CP-WX8255A; 1920 x 1080 High Definition). To produce the mirror effect, the projector was set in rear-projection mode.

A familiarization period including a set of standardized movements (touch the top of the head, touch the knees, head rotation, shoulder elevation, hip abduction) allowed subjects to explore the interaction with the avatar, while no scaling was applied. In addition, subjects were allowed to try out other spontaneous movements (e.g. walk around, jump, dance). The avatar responded to the movements of the subjects in real-time (total delay between movements and VR projection ranging between 90 and 120 ms), such that subjects felt like they were looking at themselves in a mirror. The total duration of this initial familiarization period was about 2 minutes. Subsequently, and for the entire duration of the experiment, the avatar was medially rotated 90° so that it was displayed from the side (facing left) to allow for a better view of trunk flexion-extension movements while the subjects remained faced towards the screen.

The main task consisted of performing repetitive trunk flexion-extension movements while sitting and standing. For the sitting condition, subjects were placed on a stool that was adjusted to yield 90° of hip and knee flexion. For the standing condition, subjects were instructed to keep the knee joint partially flexed. Movements always started from and ended at a static baseline position during which the trunk flexion angle was considered to be 0°. Subjects were instructed to keep their back and neck straight, their head facing the screen, arms falling naturally along the sides of the body, and feet aligned at shoulder width and pointing forward.

To make sure that they understood the task correctly, subjects were familiarized with the trunk flexion-extension task in 2 steps. First, they practiced bending forward at a slow pace and in one fluent movement towards a predefined angle of 15°, 25° or 35°. They were not informed on the degrees of flexion required or reached, but were simply instructed that required flexion angles would vary from trial to trial. When the predefined angle for a particular trial was reached (detected in D-flow based on motion capture data from 2 markers on the back of the subjects), the word “OK” appeared on the screen along with a simultaneous bell-sound. This signaled to return to the static baseline position (2 trials augmented and 2 trials reduced feedback). Second, subjects practiced the two-alternative forced choice paradigm (2 trials augmented and 2 trials reduced feedback). This meant that after each trial, subjects had to decide whether the movements of the avatar were either greater or smaller than their own movements. Notably, this required an evaluation of both visual (avatar movement) and proprioceptive (own movement) feedback. Subjects did not receive any feedback on the correctness of their response.

#### Trunk flexion task with scaled visual movement feedback

During the actual two-alternative forced choice paradigm, the visual feedback on trunk flexion-extension movements was scaled in real-time (similar scaling for both rotation amplitudes and velocities). A total number of 13 different scaling factors were used (range-0.176 to 0.176, in log-space). For negative scaling factors (< 0), the scaled rotation was reduced. For positive scaling factors (> 0), the scaled rotation was augmented. The two extremes (scaling factors-0.176 and 0.176) were only used for familiarization and test trials. These corresponded to visual feedback on movements being reduced or augmented 1.5 times and thus could be clearly perceived by the subjects. Two sets of five points (range-0.126 to 0.126) that were evenly distributed around a scaling factor of 0 (no scaling being applied) were used for analysis. The level of scaling was unknown to the subjects.

The total number of trials and maximal trunk flexion angles were kept low to ensure feasibility in all subjects, including those with CNSLBP. The number of trials was weighted per scaling factor to obtain more data for relatively difficult trials involving small modulations (i.e., trials in which the scaling factor was close to 0). Trials were distributed pseudo-randomly over 3 blocks. This ensured that each block contained a balanced number of relatively easy and relatively difficult trials. The first 2 trials of each block were test trials, and were not further analyzed. As the tasks had to be performed while sitting and standing, the total number of blocks was 6 (3 sitting and 3 standing blocks), and the total number of trials available for analysis was 126. Sitting and standing blocks were alternated and the starting block (sitting or standing) was randomized across subjects.

Different predefined flexion angles (15°, 25° and 35°) were used to ensure that proprioceptive feedback varied from trial to trial. Moreover, the 3 smallest scaling factors were not combined with a 15° flexion angle, and the 3 largest scaling factors were not combined with a 35° flexion angle to avoid extremes in the avatar’s movements. Together, this prevented subjects from basing their decision on visual feedback alone.

After each block low back pain intensity was assessed in subjects with CNSLBP using an NRS (0 = no pain, 10 = worst pain imaginable). After finishing all experimental blocks, the subjects’ perception of the interaction with the virtual mirror (immersion, distraction) was evaluated on a 1–7 scale using a set of questions from the Presence Questionnaire [34].

### Outcome parameters

For each subject, the responses to the two-alternative forced choice paradigm (greater = 1, smaller = 0) were plotted against the different scaling factors. Then a sigmoid curve (1), with initial value X_Y0.50_ = 0, with constraints Y_MAX_ = 1 and Y_MIN_ = 0, and with a variable slope (*m*), was fitted to the data (Prism 6 for Windows, Graphpad Software Inc., La Jolla, CA, USA).

$$Y\;=\;Y_{MIN}\;+\frac{Y_{MAX}-Y_{MIN}}{1+10^{\left(X_{Y0.50}-X\right)m}}$$ (1)

From each curve, 3 data points were interpolated (X_Y0.25_, X_Y0.50_, X_Y0.75_), and used to determine the point of subjective equivalence (PSE)(2) and the just noticeable difference (JND)(3).

$$PSE\;=\;X_{Y0.50}$$ (2)

$$JND\;=\;\frac{X_{Y0.75}\;-\;X_{Y0.25}}{2}$$ (3)

Theoretically, the chance distribution for a two-alternative forced choice paradigm predicts a PSE of 0, i.e. there is a 50% chance of responding “greater” or “smaller” when in fact no scaling has been applied. A PSE higher than 0 indicates that subjects overestimate their own movements. A PSE lower than 0 indicates that subjects underestimate their own movements. The higher the slope and the smaller the JND, the better subjects are able to discriminate between different levels of scaled feedback.

### Task adherence

Task adherence was assessed by analyzing maximum trunk flexion angles and maximum trunk flexion velocity using in-house scripts written in Matlab (version R2010b, The Mathworks Inc., Natik, MA, USA). Data was filtered using a second-order double pass Butterworth filter (4 Hz). Analyses of sagittal plane trunk kinematics were performed based on 3 markers located on the back of the subject (C7, T10 and scapula). In addition, we verified that there was sufficient trial-to-trial variation, confirming that proprioceptive and visual feedback varied across trials and that subjects had to depend on both visual and proprioceptive information to perform the task correctly.

### Data analysis

For each of the outcome parameters (X_Y0.25_, PSE, X_Y0.75_, JND, *m*), descriptive statistics were calculated (IBM SPSS for Windows, version 22.0.0.0, USA). Statistical testing of group differences in demographic and anthropomorphic data was performed using independent T-tests (2-tailed). For curve metrics and trunk kinematics this was performed using multivariate analyses of variance with factor [Group] (CNSLBP, HC) and post-hoc independent T-tests (2-tailed, uncorrected). Dependent variables were either [Curve metrics] (X_Y0.25_, PSE, X_Y0.75_, JND, *m*) or [Kinematics] (maximum flexion angles, maximum flexion velocity). Correlations between the PSE and CNSLBP characteristics (pain intensity, ODI, TSK, FDAQ) were explored using (uncorrected) Pearson’s correlations.

## Results

Of the total of 32 subjects that participated in the experiment, two subjects (one subject with CNSLBP and one HC subject) showed poor task adherence (regarding instructions to move slowly and in one fluent movement) and were also identified as outliers (data outside 1.5 times the interquartile range) regarding psychophysical curve metrics and kinematics. As such these subjects were excluded from the analyses. The final sample therefore consisted of 30 male subjects, with 15 subjects in each group. Demographic and anthropomorphic data are presented in Table 1 and pain characteristics in Table 2.

**Table 1:** Demographic and anthropomorphic data, mean ± SD.

	CNSLBP (n = 15)	HC (n = 15)	p
Age (years)	36 ± 10	34 ± 9	0.581
Weight (kg)	88 ± 19	91 ± 13	0.699
Height (cm)	176 ± 7	176 ± 9	0.854
BMI	28 ± 5	29 ± 4	0.498

BMI: body mass index, CNSLBP: chronic non-specific low-back pain, HC: healthy control, p: p-value for statistical testing (independent T-test, 2-tailed).

**Table 2:** CNSLBP characteristics.

Pain characteristic	CNSLBP (n = 15)
Time since onset of current LBP episode (months)	12 (1–180)
Total number of episodes experienced (n)	2 (1–10)
Pain frequency (number of days per week)	6 (1–7)
Pain location	
Center / Right / Left	4 / 1 / 0
Combination	10
Average pain intensity over the last 48h (NRS: 0–100)	30 (0–50)
Maximum pain intensity over the last 48h (NRS: 0–100)	40 (10–80)
LBP related disability (ODI: 0%-100%)	24 (10–44)
Activities increasing pain	
Standing (prolonged)	14
Sitting (prolonged)	11
Trunk flexion	10
Kinesiophobia	
General (TSK: 17–68)	39 (24–55)
Daily activities (FDAQ: 0–100)	27 (3–68)
Treatment	
Physiotherapy	13
Analgesics	6
Antidepressants	3
Co-morbidities	
Upper back/neck pain	4
Lower-limb pain	6
Post-traumatic stress syndrome	4

Data are presented as median (min-max) or as number of subjects. CNSLBP: chronic non-specific low-back pain, FDAQ: fear of daily activities questionnaire, ODI: Oswestry disability index, TSK: Tampa scale of kinesiophobia.

Groups were statistically equivalent for age, height, weight, and BMI. For subjects with CNSLBP, reported pain intensity was relatively low and disability (ODI) could be classified as minimal (n = 7) to moderate (n = 8). Most of them (n = 13) could be classified as having kinesiophobia based on a score of at least 37 on the TSK. Pain intensity as assessed between experimental blocks did not exceed the usual variability in pain intensity reported over the previous 48h. Of the 15 subjects with CNSLBP, 11 had work restrictions related to CNSLBP and/or to co-morbidities (4 permanent, 7 temporary).

### Perception of scaled trunk movements

**Fig 1:**
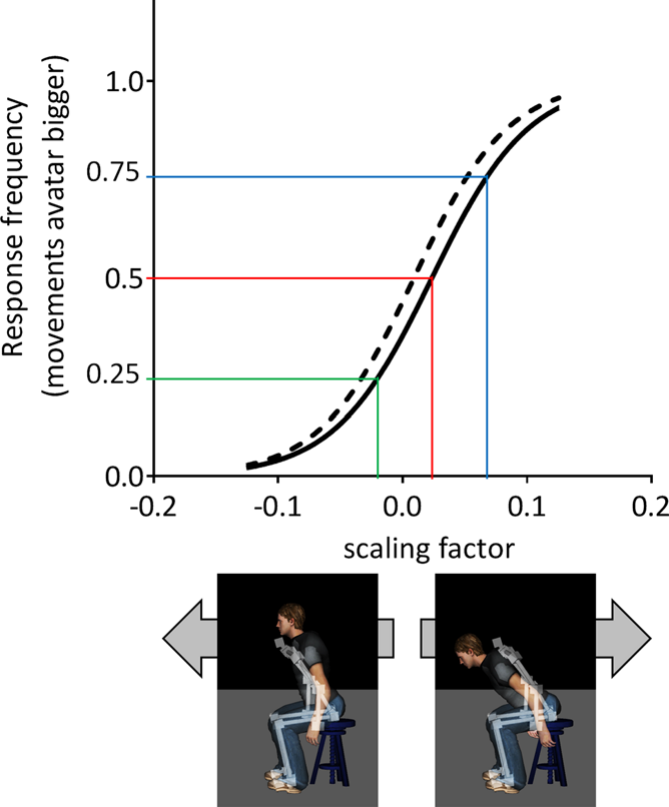
Grand average psychophysical curves for subjects with CNSLBP (black line) and HC subjects (black interrupted line). Colored lines indicate the curve metrics derived: X_Y0.25_ (green), X_Y0.50_ = point of subjective equality (PSE) (red), and X_Y0.75_ (blue). CNSLBP: chronic non-specific low-back pain, HC: healthy control. A PSE higher than 0 indicates that subjects with CNSLBP tended to overestimate their own movements. The two screenshots and arrows illustrate the effect of negative (left) and positive (right) scaling factors on avatar movements based on actual movements (white skeleton).

Curve metrics are presented in Table 3 and Fig. 1. Overall, groups presented with similar curve metrics (F_3,26_ = 1.319, p = 0.289). The lack of differences between groups for the JND and *m*, indicates that the ability to discriminate between different levels of scaling was comparable between groups. However, there was a trend towards higher values for the PSE (p = 0.061) and for the X_Y0.75_ (p = 0.081) in subjects with CNSLBP as compared to HC subjects. Moreover, the 95% confidence interval of the PSE in subjects with CNSLBP did not include 0, whereas it did in HC subjects. As such, it appears that the psychophysical curve is somewhat shifted to the right in subjects with CNSLBP, indicating a perceptual bias towards overestimating their own movements. No significant correlations were observed between the PSE and CNSLBP characteristics.

**Table 3:** Summary of curve metrics, mean (95% CI).

	CNSLBP (n = 15)	HC (n = 15)	p
X_Y0.25_	-0.016	-0.030	0.154
	(-0.029; -0.002)	(-0.0448; -0.0146)	
PSE	0.027	0.008	0.061
	(0.010; 0.044)*	(-0.0049; 0.0210)	
Y_0.75_	0.070	0.046	0.081
	(0.043; 0.096)	(0.0334; 0.0581)	
JND	0.043	0.038	0.420
	(0.031; 0.054)	(0.033; 0.042)	
*m*	13.3	13.4	0.975
	(10.5; 16.2)	(11.6; 15.0)	

Data are presented as mean (95% confidence interval, lower bound; upper bound). CNSLBP: chronic non-specific low-back pain, HC: healthy control, *m*: slope, p: p-value for statistical testing (independent T-test, 2-tailed), PSE: point of subjective equality, X_Y0.25_: interpolated log scaling factor at a response frequency of 0.25, Y_0.75_: interpolated log scaling factor at a response frequency of 0.75.

*in contrast to HC, the confidence interval of the PSE for subjects with CNSLBP does not include 0.

### Task adherence

Overall, task adherence was adequate and groups presented with similar movement kinematics (F_3,26_ = 1.709, p = 0.190). Mean flexion angles ± SD were slightly smaller in subjects with CNSLBP (34 ± 5 degrees) as compared to HC subjects (37 ± 4) (p = 0.043), which is consistent with the slightly higher maximum flexion velocities (leading to increased reaction times following the appearance of the “OK” sign and simultaneous bell-sound) observed in HC subjects (CNSLBP: 26 ± 14 degrees/s; HC: 32 ± 8 degrees/s; p = 0.171).

### VR immersion

Overall ratings for VR immersion and distraction are presented in Table 4. VR immersion was generally reported to be very good (i.e., average ratings higher than 5), despite some perceived delays and despite the fact that the subject’s mirror image was presented in side-view mode. Distraction due to the visual display and/or control devices was considered minimal. This indicates that immersion issues (if any) due to the presentation of the mirror image in side-view mode were adequately prevented (i.e. by familiarizing the subjects in mirror mode on forehand) and/or resolved (i.e. by practicing the task on forehand) prior to the start of the actual experiment.

**Table 4:** VR immersion.

	Question	n = 30
I	How much were you able to control the avatar (your virtual image)?	6.1 ± 0.8
	(1 = not at all, 7 = completely)	
	How responsive was the avatar to your movements?	6.0 ± 0.7
	(1 = not responsive, 7 = completely responsive)	
	How quickly did you adjust to the virtual environment experience?	6.3 ± 1.1
	(1 = not at all, 7 = less than 1 minute)	
	How proficient in moving and interacting with the virtual environment did you feel at the end of the experience?	6.2 ± 0.8
	(1 = not proficient, 7 = very proficient)	
	To what extent did the movements of the avatar seem natural to you?	5.6 ± 0.8
	(1 = extremely artificial, 7 = completely natural)	
	How well could you examine the details of the avatar?	5.6 ± 1.0
	(1 = not at all, 7 = extensively)	
D	How much delay did you experience between your actions and the response of the system?	2.5 ± 1.6
	(1 = no delays, 7 = long delays)	
	How much did the visual display quality interfere or distract you from performing assigned tasks or required activities?	1.7 ± 1.0
	(1 = not at all, 7 = prevented task performance)	
	How much did the control devices interfere with the performance of assigned tasks or with other activities?	1.2 ± 0.4
	(1 = not at all, 7 = interfered greatly)	

Data are presented as mean ± SD. I: immersion, D: distraction.

## Discussion

In this study a “virtual mirror” was combined with a trunk flexion task to assess body perception during active movement in military personnel with CNSLBP as compared to military HC subjects. It was shown that despite a similar ability to discriminate between different levels of scaling, subjects with CNSLBP tended to overestimate their own movements, as evidenced by a right-shifted psychophysical curve.

### Body perception assessment

Body perception, for example relating to the size of body parts or to the position of body parts in space, is dependent on a comparison of multimodal sensory inputs including visual, haptic, proprioceptive, and vestibular information [35,36,37], and can occur consciously or unconsciously. As introduced earlier, a variety of different methods can be used to study different aspects of body perception and its disturbances. The task used in the present study had two important characteristics. First, it assessed body perception implicitly, i.e. no explicit instruction was given to actually try and perceive the body (for example such as described by [2]). Second, as body perception was assessed during active movement, it required a continuous comparison between sensory inputs and motor output [38]. As such, our task allowed for an assessment of body perception that was not possible using previous methods, making it particularly relevant for the assessment of patient populations presenting with clinical signs of body perception disturbances, movement-related pain and/or movement dysfunctions. Importantly, the methodology applied in this study need not to be limited to trunk flexion movements but could also be applied to other types of body or limb movements. Furthermore, this methodology could be used to apply prolonged periods of augmented or reduced visual movement feedback to study adaptation processes [35] or as an intervention to normalize body perception disturbances, abnormal movement patterns and pain [1,5].

### Body perception and pain

The present study assessed body perception in the presence of CNSLBP. Previous work in patients with CNSLBP from the general population showed that disturbances in body perception, assessed explicitly by asking patients how they perceived their back, overlapped with decreases in tactile acuity and with the distribution of clinical pain [12]. Other studies showed that patients with CNSLBP rely less on back muscle proprioception during postural control [3,25], and that this could be reinforced by visual deprivation (i.e., by reducing exteroceptive input) [23]. As such, visual information may in part compensate for some of the interoceptive impairments observed in patients with CNSLBP [39]. Still, several studies have also shown that visual information processing itself could be impaired in CNSLBP. For example, Bowering et al. (2014) showed that patients with a history of low back pain performed worse than healthy controls on a task involving the visual judgment of the laterality of back-images [15]. Also, de Lussanet et al. (2013) showed that patients with CNSLBP were selectively impaired in visually judging the manipulation of weights involving movements associated with clinical pain (i.e., trunk-rotation movements) [22]. Together, these studies suggest that impaired body perception in patients with CNSLBP could be due to impaired interoception, impaired exteroception, or a combination of both.

In contrast to previous work, the present study in military personnel did not find actual impairments in body perception, since subjects with CNSLBP and HC subjects showed a similar ability to discriminate between different levels of scaling (JND and slope of the psychophysical curve). Still, subjects with CNSLBP showed a performance bias and tended to overestimate their own movements (right-shifted psychophysical curve). Such perceptual bias has generally not been assessed in previous work, but was found to be absent in a group of patients with CNSLBP during a simple visual judgment of movements (performed while the own body remained static) [22]. Therefore, the present results extend previous work on the relationship between body perception and pain in patients with CNSLBP. In addition, they confirm the notion that body perception disturbances during active movement could be different from those under static conditions, and could be associated with different underlying neurophysiological mechanisms. As such, patients presenting with body perception disturbances during active movement might require different clinical management than those presenting with body perception disturbances under static conditions.

Yet, only a trend towards abnormal body perception was observed when directly comparing psychophysical curve metrics between subjects with CNSLBP and HC subjects. In addition to the obvious limitations of an exploratory study (e.g. small sample size, potential confounding due to co-morbidities/medication), this might be explained by the relatively low range in pain intensities and disability levels reported by the subjects with CNSLBP enrolled in this study, which may have reduced the contrast with HC subjects. For example, in previous studies assessing patients with CNSLBP from the general population, average pain intensities were reported in the range of 50–60 on a scale of 100 [12,22]. Still, we were hesitant to apply more strict inclusion criteria in terms of minimal pain level as, like endurance athletes [40], military personnel may perceive and report pain and disability differently as compared to people from the general population. This is supported by another recent study in military personnel (n = 11) that also reported relatively mild CNSLBP (average pain intensity of 45/100, average ODI of 17/100), despite having employed more strict inclusion criteria (pain intensity ≥ 30/100, ODI ≥ 12/100) [41].

Another factor that should be taken into account is that military work generally requires high levels of physical activity. Indeed, most subjects with CNSLBP enrolled in this study were performing physical exercise on a daily basis, despite pain. This contrasts with reduced levels of physical activity reported for patients with CNSLBP as compared to healthy controls from the general population [24]. The relatively mild presentations of CNSLBP and relatively high levels of physical activity reported in our population of military personnel may also explain why no correlations were found between psychophysical curve metrics and CNSLBP characteristics. In addition, this could explain why, in contrast to reports on patients with CNSLBP from the general population [7], only small differences were observed when comparing kinematics between our subjects with CNSLBP and HC subjects. Still, given the observed trend towards abnormal body perception in mildly affected military personnel with CNSLBP, our method is expected to have sufficient sensitivity to detect body perception disturbances in patients from the general population or in military personnel with more severe disability. The next step will therefore be to assess a larger sample of patients with a more heterogeneous clinical profile to further explore relationships between clinical characteristics and body perception during active movement. In addition, it would be interesting to further assess the relationship between body perception during active movement and other aspects of body perception (e.g. during static conditions), or in relation to feelings of ownership over the virtual mirror image, which could be different in patients with chronic pain [42]. Lastly, associations between abnormal body perception and the presence of CNSLBP during movements in other than the sagittal plane, remain to be confirmed in future studies.

## Conclusions

This study investigated body perception during active movement and showed that despite a similar ability to discriminate between different levels of scaling, military personnel with CNSLBP tended to overestimate their own movements as compared to military HC subjects. These results denote an important relationship between body perception, movement and pain, and warrant further assessment of the neurophysiological mechanisms involved. Moreover, this study showed that a scalable “virtual mirror” might assist in the assessment of body perception during active movement, which offers new avenues for understanding and managing the interaction between body perception disturbances and abnormal movement patterns in patients with pain.
